# Effect of Normal Level Endocrine Hormones and Hypothalamic Neuropeptides on Obesity in Women of Childbearing Age

**DOI:** 10.1155/jobe/4561022

**Published:** 2026-03-13

**Authors:** Yingying Tang, Xiukun Zhang, Lei Fan, Chengyuan Li, Yudong Guo, Fuman Du, Weimin Wang

**Affiliations:** ^1^ Department of Endocrinology, Heilongjiang Provincial Hospital, Harbin, Heilongjiang, China, hewiki.com; ^2^ Department of Orthopaedics, The First Affiliated Hospital of Heilongjiang University of Traditional Chinese Medicine, Harbin, Heilongjiang, China; ^3^ Department of Gastroenterology, Heilongjiang Provincial Hospital, Harbin, Heilongjiang, China, hewiki.com; ^4^ Cardiovascular Disease Center, Heilongjiang Provincial Hospital, Harbin, Heilongjiang, China, hewiki.com

**Keywords:** childbearing age, endocrine hormones, female, neuropeptide, obesity

## Abstract

**Aims:**

To explore the association of endocrine hormones and hypothalamic neuropeptides fluctuations within normal level with obesity in women of childbearing age.

**Materials and Methods:**

Laboratory data of women in childbearing age with normal levels of endocrine hormones and hypothalamic neuropeptides in Heilongjiang Provincial Hospital between March 2022 and October 2022 were retrospectively collected. Included subjects were divided into obese group and normal weight group according to body mass index.

**Results:**

80 subjects were included in final analysis, including 37 in the obese group and 43 in the normal weight group. Within the normal range, the levels of cortisol, adrenocorticotropic hormone, free triiodothyronine, free thyroxine, follicle‐stimulating hormone, neuropeptide Y, and orexin were positively correlated with body mass index, while the levels of estradiol and oxytocin were negatively correlated with body mass index; the levels of adrenocorticotropic hormone, thyroid‐stimulating hormone, neuropeptide Y, and orexin were positively correlated with triglycerides, while proopiomelanocortin level was negatively correlated with triglycerides; neuropeptide Y level was positively correlated with total cholesterol, and cortisol level was negatively correlated with high‐density lipoprotein cholesterol. Elevated levels of neuropeptide Y and orexin were independent risk factors for obesity (OR = 1.123, 95% CI = 1.023–1.232, *p* = 0.015; OR = 4.004, 95% CI = 1.174–13.656, *p* = 0.027), while increase in oxytocin level was an independent protective factor (OR = 0.833; 95% CI = 0.697–0.995; *p* = 0.044).

**Conclusions:**

Among women of childbearing age, fluctuations in multiple endocrine hormones and hypothalamic neuropeptides within the normal range play a crucial role in regulating body weight and plasma lipids. Specifically, relatively higher levels of neuropeptide Y and orexin increase obesity risk, while elevated oxytocin levels reduce this risk. These identified biomarkers may offer candidates for subsequent mechanistic research and clinical interventions.

## 1. Introduction

In recent years, obesity has become a serious public health problem worldwide, and the prevalence of obesity is on the rise [1]. It is expected that by 2025, obesity will affect about 20% of the world’s population, with the prevalence of obesity reaching 18% in men and 21% in women [2]. Overweight and obesity are very prevalent among women of childbearing age, with 30%–40% of women in their reproductive years in developing countries, and many large cohort studies have shown that this age group is the period of greatest weight gain [3, 4]. Women of childbearing age are the key population for the prevention of obesity and metabolic diseases who are more susceptible to various complications of pregnancy and childbirth, and the risk of birth defects and death of newborns are significantly increased [1].

At present, the mechanism of obesity has not been fully elucidated. Its development is modulated by a complex interplay of physiological, psychological, behavioral, familial, social, cultural, and environmental factors, as well as genetic susceptibility. For women of childbearing age, modern sedentary lifestyles and insufficient sleep—attributed to chronic stress—can increase cortisol secretion, disrupt circadian rhythms, and impair endocrine homeostasis. These perturbations further alter eating behaviors, appetite regulation, and nutrient absorption, collectively contributing to the elevated prevalence of obesity [1, 5]. Additionally, comorbidities such as hypothyroidism, polycystic ovary syndrome, and metabolic syndrome may also exacerbate obesity development [1].

The endocrine gland axis plays a crucial role in the process of feeding, energy metabolism, and weight regulation, including the hypothalamic–pituitary–adrenal axis, the hypothalamic–pituitary–thyroid axis, and the hypothalamic–pituitary–gonadal axis. Besides, a variety of neuropeptides expressed by hypothalamic neurons are closely related to feeding behavior [6–8]. Disruptions in the secretion of these endocrine hormones and central nervous peptides may be involved in the development of obesity. Most studies focused on people with abnormal endocrine gland axis function, who have relatively more pronounced indicators of obesity‐related metabolic disorders. The purpose of this study was to explore whether endocrine hormones and hypothalamic neuropeptides within the normal level contributed to the development of obesity in women of childbearing age.

## 2. Materials and Methods

The demographic data and obesity‐related laboratory indicators of women aged 18 to 45 years were collected from the Department of Endocrinology of Heilongjiang Provincial Hospital between March 2022 and October 2022. Subjects with normal levels of endocrine hormones and hypothalamic neuropeptides were included. Those with one or more of the following conditions were excluded: body mass index (BMI) < 18.5 kg/m^2^ or 23.9 < BMI < 28 kg/m^2^, diabetes, thyroid disease, adrenal gland disease, pituitary disease, gonadal disease, malignant tumor, psychiatric disease, severe gastrointestinal tract disease, heart failure, severe kidney disease, took hormonal drugs (including oral contraceptives) or immunomodulators in the past two months, irregular menstrual cycles, and pregnant or lactating. According to BMI, subjects were divided into obese group (≥ 28.0 kg/m^2^) and normal weight group (18.5–23.9 kg/m^2^). The general clinical data (sex, age, height, weight, marital status, smoking history, and alcohol history) and laboratory data including total cholesterol, triglycerides, low‐density lipoprotein cholesterol, high‐density lipoprotein cholesterol, serum cortisol, adrenocorticotropic hormone, free triiodothyronine, free thyroxine, thyroid‐stimulating hormone, testosterone, progesterone, estradiol, luteinizing hormone, follicle‐stimulating hormone, prolactin, serum neuropeptide Y, proopiomelanocortin, orexin, and oxytocin were extracted from medical record system by trained research assistants.

Total cholesterol, triglycerides, low‐density lipoprotein cholesterol, and high‐density lipoprotein cholesterol were examined in the Biochemistry Laboratory of the Experimental Diagnosis Department of Heilongjiang Provincial Hospital using a fully automatic biochemical analyzer (Hitachi, Ltd., Tokyo, Japan). Cortisol, adrenocorticotropic hormone, free triiodothyronine, free thyroxine, thyroid‐stimulating hormone, testosterone, progesterone, estradiol, luteinizing hormone, follicle‐stimulating hormone, and prolactin were examined by electrochemiluminescence immunoassay analyzer (Roche Diagnostics, Mannheim, Germany) in the Department of Radioimmunology. Serum neuropeptide Y, proopiomelanocortin, orexin, and oxytocin were examined with enzyme‐linked immunosorbent assay (ELISA kit) from Shanghai Tianhao Biotechnology Co., Ltd. All blood samples were collected in the morning between 8:00 a.m. and 10:00 a.m. on an empty stomach (with an 8‐h fasting period). Additionally, all participants were instructed to avoid strenuous physical activity or emotional excitement on the day of sampling. The study was conducted in accordance with the Declaration of Helsinki and approved by the Institutional Review Board of Heilongjiang Provincial Hospital [2022(102)]. The need for additional written informed consent was waived due to the retrospective nature of the study.

### 2.1. Definition

According to the Guidelines for the Prevention and Control of Overweight and Obesity in Chinese Adults, a BMI of 18.5–23.9 kg/m^2^ is considered normal weight, a BMI of 24.0–27.9 kg/m^2^ is considered overweight, and a BMI of ≥ 28.0 kg/m^2^ is considered obese. Smoking history was defined as an average of one cigarette per day for more than 1 year, or less than 1 year of abstinence. Alcohol history was defined as alcohol consumption for more than 5 years, 20 g/d for women.

### 2.2. Statistical Analysis

SPSS 27.0 was used for data analysis. Exploratory data analysis and Shapiro–Wilk tests were performed to determine the normality of the data distribution. Normally distributed continuous data were expressed as the mean ± standard deviation and compared using Student’s *t* test. Nonnormally distributed continuous data were expressed as median (M) and interquartile range (IQR) and compared using Mann–Whitney *U* test. Categorical variables were presented as counts and percentages and compared using the Pearson *χ* ^2^ test. Correlation analysis was performed using Spearman correlation analysis. A binary logistic regression analysis was performed to identify risk factor for obesity, and each odds ratio (OR) was calculated with a 95% confidence interval (CI). *p* value of < 0.05 was considered statistically significant, and all tests were two‐sided.

## 3. Results

### 3.1. Demographic Characteristics and Plasma Lipid Levels

**FIGURE 1:**
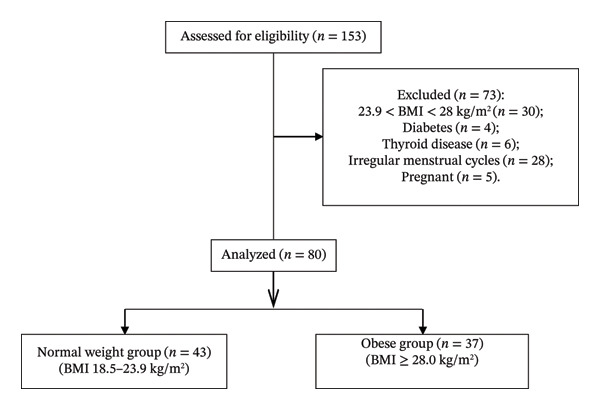
Participant flow diagram. BMI, body mass index.

A total of 80 subjects were included in final analysis (Figure 1), including 37 in the obese group (mean age 42, range 26∼44 years old) and 43 in the normal weight group (mean age 38, range 18∼44 years old). As shown in Table 1, the mean age, proportions of smoking history, alcohol history, and marital status in the obese group were significantly higher than those in the normal weight group (*p* < 0.05).

**TABLE 1:** Demographic characteristics and plasma lipid levels.

Variables	Obese group (*n* = 37)	Normal weight group (*n* = 43)	*p*
BMI (kg/m^2^)	29.00 (2)	21.30 (3)	< 0.001
Age (years)	42.00 (7)	38.00 (12)	0.046
Smoking history, *n* (%)	10 (27.0)	3 (7)	0.03
Alcohol history, *n* (%)	18 (48.6)	1 (2.32)	< 0.001
Marital status, *n* (%)	37 (100)	37 (86)	0.028
Total cholesterol (mM)	5.40 (1)	4.97 (1)	0.157
Triglycerides (mM)	1.90 (2)	0.98 (1)	< 0.001
Low‐density lipoprotein cholesterol (mM)	2.88 ± 0.691	2.70 ± 0.847	0.297
High‐density lipoprotein cholesterol (mM)	1.31 (0)	1.45 (1)	0.057

*Note:* BMI, age, levels of total cholesterol, triglycerides, and high‐density lipoprotein cholesterol were expressed as M (IQR) and compared using Mann–Whitney *U* test.

Abbreviation: BMI, body mass index.

### 3.2. Levels of Endocrine Hormones and Hypothalamic Neuropeptides

There was no significant difference in the levels of free thyroxine, thyroid‐stimulating hormone, prolactin, testosterone, progesterone, and luteinizing hormone between the two groups (*p* > 0.05). The levels of cortisol, adrenocorticotropic hormone, free triiodothyronine, follicle‐stimulating hormone, neuropeptide Y, and orexin in the obese group were significantly higher than those in the normal weight group (*p* < 0.05), while the levels of estradiol, proopiomelanocortin, and oxytocin were significantly lower than those in the normal weight group (*p* < 0.05) (Table 2).

**TABLE 2:** Comparison of levels of endocrine hormones and hypothalamic neuropeptides.

Variables	Obese group (*n* = 37)	Normal weight group (*n* = 43)	*p*
Free triiodothyronine (pM)	3.94 ± 0.717	3.46 ± 0.839	0.008
Free thyroxine (pM)	13.73 ± 1.909	13.20 ± 1.849	0.211
Prolactin (ng/mL)	10.04 (6)	12.97 (8)	0.077
Testosterone (ng/mL)	0.30 ± 0.107	0.30 ± 0.083	0.853
Progesterone (ng/mL)	0.19 (0)	0.24 (4)	0.189
Luteinizing hormone (mIU/mL)	4.18 (11)	4.05 (3)	0.589
Cortisol (μg/dL)	16.89 (11)	8.81 (5)	< 0.001
Adrenocorticotropic hormone (pg/mL)	38.56 (24)	23.31 (15)	< 0.001
Thyroid‐stimulating hormone (mIU/L)	2.13 (2)	1.64 (2)	0.359
Follicle‐stimulating hormone (mIU/mL)	5.43 (10)	4.46 (3)	0.005
Neuropeptide Y (ng/mL)	224.90 ± 29.735	175.92 ± 24.932	< 0.001
Orexin (ng/mL)	12.74 (4)	8.38 (4)	< 0.001
Estradiol (pM)	38.53 (25)	68.60 (68)	0.004
Proopiomelanocortin (pg/mL)	6125.35 (1138)	6426.65 (1582)	0.003
Oxytocin (pg/mL)	110.14 (39)	146.49 (59)	< 0.001

*Note:* Levels of prolactin, progesterone, luteinizing hormone, cortisol, adrenocorticotropic hormone, thyroid‐stimulating hormone, follicle‐stimulating hormone, orexin, estradiol, proopiomelanocortin, and oxytocin are expressed as M (IQR) and compared using Mann–Whitney *U* test.

### 3.3. Logistic Regression Analysis of Influencing Factors for Obesity in Women of Childbearing Age

The multivariate regression model was performed with forward method in consideration of collinearity existed among the variables. As shown in Table 3, elevated levels of neuropeptide Y and orexin were independent risk factors for obesity (OR = 1.123, 95% CI = 1.023–1.232, *p* = 0.015; OR = 4.004, 95% CI = 1.174–13.656, *p* = 0.027), while higher level of oxytocin was an independent protective factor (OR = 0.833; 95% CI = 0.697–0.995; *p* = 0.044).

**TABLE 3:** Independent variables associated with obesity in multivariate regression analysis.

	*B*	*P*	OR	95% CI
Neuropeptide Y	0.116	0.015	1.123	1.023–1.232
Orexin	1.387	0.027	4.004	1.174–13.656
Oxytocin	−0.183	0.044	0.833	0.697–0.995

### 3.4. Correlation Analysis of Endocrine Hormones and Hypothalamic Neuropeptides With BMI and Plasma Lipid Levels

Endocrine hormone: Cortisol level was positively correlated with BMI (*r* = 0.432, *p* < 0.001) and negatively correlated with high‐density lipoprotein cholesterol level (*r* = −0.283, *p* = 0.011). Adrenocorticotropic hormone level was positively correlated with BMI and triglyceride level (*r* = 0.307, *p* = 0.006; *r* = 0.290, *p* = 0.009). Levels of free triiodothyronine and free thyroxine were positively correlated with BMI (*r* = 0.325, *p* = 0.003; *r* = 0.221, *p* = 0.049). Thyroid‐stimulating hormone level was positively correlated with triglyceride level (*r* = 0.319, *p* = 0.004). Estradiol level was negatively correlated with BMI (*r* = −0.320, *p* = 0.004). Follicle‐stimulating hormone level was positively correlated with BMI (*r* = 0.233, *p* = 0.037) (Tables 4, 5, and 6).

**TABLE 4:** Correlation analysis of hypothalamic–pituitary–adrenal axis hormones with BMI and plasma lipid levels.

Variables	Cortisol		Adrenocorticotropic hormone	
	*r*	*P*	*r*	*P*
BMI (kg/m^2^)	0.432	< 0.001	0.307	0.006
Total cholesterol (mM)	−0.028	0.808	0.034	0.765
Triglyceride (mM)	0.200	0.076	0.290	0.009
High‐density lipoprotein cholesterol (mM)	−0.283	0.011	−0.145	0.199
Low‐density lipoprotein cholesterol (mM)	0.037	0.747	−0.029	0.795

Abbreviation: BMI, body mass index.

**TABLE 5:** Correlation analysis of hypothalamic–pituitary–thyroid axis hormones with BMI and plasma lipid levels.

Variables	Free triiodothyronine		Free thyroxine		Thyroid‐stimulating hormone	
	*r*	*P*	*r*	*P*	*r*	*P*
BMI (kg/m^2^)	0.325	0.003	0.221	0.049	0.053	0.640
Total cholesterol (mM)	0.059	0.601	−0.087	0.444	−0.025	0.829
Triglyceride (mM)	0.084	0.459	−0.155	0.171	0.319	0.004
High‐density lipoprotein cholesterol (mM)	0.001	0.996	0.023	0.838	−0.187	0.097
Low‐density lipoprotein cholesterol (mM)	0.003	0.982	−0.166	0.142	0.033	0.774

Abbreviation: BMI, body mass index.

**TABLE 6:** Correlation analysis of hypothalamic–pituitary–gonadal axis hormones with BMI and plasma lipid levels.

**Variables**	**Prolactin**		**Testosterone**		**Progesterone**	
	***r***	***P***	***r***	***P***	***r***	***P***

BMI I (kg/m^2^)	−0.091	0.424	0.026	0.819	−0.081	0.477
Total cholesterol (mM)			0.000	0.999	0.121	0.287
Triglyceride (mM)	−0.041	0.715	−0.039	0.730	−0.035	0.756
High‐density lipoprotein cholesterol (mM)	−0.050	0.662	−0.121	0.283	−0.014	0.904
Low‐density lipoprotein cholesterol (mM)	0.055	0.628	0.043	0.702	0.123	0.279

**Variables**	**Luteinizing hormone**		**Follicle-stimulating hormone**		**Estradiol**	
	***r***	***P***	***r***	***P***	***r***	***P***

BMI (kg/m^2^)	−0.045	0.694	0.233	0.037	−0.320	0.004
Total cholesterol (mM)	0.013	0.908	−0.125	0.269	0.090	0.426
Triglyceride (mM)	0.140	0.215	0.191	0.090	−0.115	0.311
High‐density lipoprotein cholesterol (mM)	−0.150	0.185	−0.195	0.082	0.098	0.388
Low‐density lipoprotein cholesterol (mM)	0.129	0.253	0.025	0.827	0.090	0.427

Abbreviation: BMI, body mass index.

Hypothalamic neuropeptide: Neuropeptide Y level was positively correlated with BMI, triglycerides level, and total cholesterol level (*r* = 0.521, *p* < 0.001; *r* = 0.376, *p* < 0.001; *r* = 0.276, *p* = 0.013). Proopiomelanocortin level was negatively correlated with triglycerides level (*r* = −0.269, *p* = 0.016). Orexin level was positively correlated with BMI and triglycerides level (*r* = 0.498, *p* < 0.001; *r* = 0.320, *p* = 0.004). Oxytocin level was negatively correlated with BMI (*r* = −0.615, *p* < 0.001) (Table 7).

**TABLE 7:** Correlation analysis of hypothalamic neuropeptides level with BMI and plasma lipid levels.

Variables	Neuropeptide Y		Orexin		Oxytocin		Proopiomelanocortin	
	*r*	*P*	*r*	*P*	*r*	*P*	*r*	*P*
BMI (kg/m^2^)	0.521	< 0.001	0.498	< 0.001	−0.615	< 0.001	−0.200	0.075
Total cholesterol (mM)	0.276	0.013	0.191	0.090	−0.128	0.257	−0.141	0.212
Triglyceride (mM)	0.376	< 0.001	0.320	0.004	−0.151	0.180	−0.269	0.016
High‐density lipoprotein cholesterol (mM)	−0.150	0.185	−0.076	0.506	0.047	0.681	−0.005	0.965
Low‐density lipoprotein cholesterol (mM)	0.168	0.136	0.098	0.387	−0.066	0.558	−0.044	0.697

Abbreviation: BMI, body mass index.

## 4. Discussion

In this study, the correlation between obesity in women of childbearing age and a variety of hormones in the endocrine gland axis and hypothalamic neuropeptides within the normal range was analyzed, proving a reference for the etiological research and intervention treatment of obesity in women during their special period.

Studies have found that hypothalamic–pituitary–adrenal axis hyperfunction is directly related to many metabolic diseases, such as obesity, hyperglycemia, dyslipidemia, insulin resistance, and metabolic syndrome [9]. The results of this study showed that the levels of cortisol and adrenocorticotropic hormone within the normal range were significantly higher in obese women than those in women of normal weight and were positively correlated with BMI and triglycerides level. The results demonstrated that cortisol and adrenocorticotropic hormone played a role in regulating body weight and plasma lipids in women during the reproductive period, and mild hyperactivity of the adrenal axis can also lead to obesity and dyslipidemia even within their normal levels. This is similar to the results in animal models, which found that relatively high level of cortisol in the case of normal adrenal cortical function or in vitro corticosterone injections led to obesity, and triglycerides level was lowered in obese rodents with partial adrenal resection that maintained normal adrenal cortex [10–12]. High cortisol levels, often associated with chronic stress, can increase the production of these reactive oxygen species, potentially leading to cellular damage and contributing to various health problems on tissues. This process is tightly controlled by a negative feedback loop where rising cortisol levels inhibit further release of corticotropin‐releasing hormone and adrenocorticotropic hormone, thus maintaining a balance. Obesity can induce systemic oxidative stress through several biochemical mechanisms, and oxidative stress may in turn aggravate adipocyte damage and metabolic abnormalities, forming a vicious circle [13–15].

Thyroid hormones play a crucial role in the differentiation of adipocytes, which in turn affects the body’s energy metabolism. The results of this study showed that within the normal range, free triiodothyronine level was significantly higher than that in the normal weight group, and thyroid hormones were positively correlated with BMI and triglycerides level, indicating that subtle changes in thyroid function also might affect the body’s metabolism and led to obesity. Previous researches on the role of hypothalamic–pituitary–thyroid axis hormone in adjusting body weight showed that thyroid hormones regulate adipocyte differentiation and energy metabolism, and even slight fluctuations may affect body weight [16, 17]. However, most of these studies have focused on people with abnormal thyroid function, who had relatively more pronounced indicators of obesity‐related metabolic disorders.

The results of this study showed that the level of estradiol within the normal range was significantly lower in obese women than in women of normal weight and was inversely correlated with BMI. Sex hormones are important regulators of mammalian food intake and energy balance, interacting with gastrointestinal hormones and central neurotransmitters to achieve central control of appetite and metabolism. Our results were in consistent with those in animal models, which found that exogenous estradiol supplementation increased the total body oxygen and energy expenditure of mice with oophorectomy [18], and estradiol level was negatively correlated with triglycerides, total cholesterol, and low‐density lipoprotein cholesterol, while positively correlated with high‐density lipoprotein cholesterol [19]. Besides, a positive correlation between follicle‐stimulating hormone level and BMI was observed in this study. Animal experiment indicated that inhibition of endogenous follicle‐stimulating hormone and blockade of its signaling pathway could reduce body fat and serum cholesterol in mice [20–22]. The findings provided a new idea for weight loss in obese women with menopausal hypercholesterolemia.

Neuropeptide Y is an appetite‐promoting factor [23]. The results of this study showed that the neuropeptide Y level in the obese group was significantly higher than that in the normal‐weight group, and it was positively correlated with BMI and levels of triglycerides and total cholesterol; meanwhile, neuropeptide Y was an independent risk factor for obesity. The findings suggested that a relatively high neuropeptide Y level within the normal range may increase the risk of obesity in women of childbearing age by promoting appetite and lipid synthesis. Animal experiments showed that increased central neuropeptide Y level in rodents by intraventricular injection led to intense feeding and the development of morbid obesity, and intraventricular injection of neuropeptide Y antagonists decreased fat storage, increased GDP‐binding activity of brown adipocytes and caloric expenditure, and improved lipodystrophy [24–26]. Investigation on neuropeptide Y‐related receptor agonists and antagonists may be the target of future obesity control. In addition, in an experiment on the effect of neuropeptide Y deficiency on fat metabolism in male and female mice, it was found that estradiol level was negatively correlated with neuropeptide Y level, and neuropeptide Y deficiency could activate estradiol‐mediated thermogenesis to reduce obesity in female mice, but not to alter serum estradiol levels and obesity in male rats, suggesting that neuropeptide Y was more closely related to the regulation of women’s body weight [27].

Currently, orexin has been found to be involved in human appetite regulation, energy balance, sleep, and wake regulation [28]. The results of this study showed that the orexin level within normal range in obese women was significantly higher than that in women of normal weight and was positively correlated with BMI and triglycerides level. The findings demonstrated that higher level of orexin within normal range might lead to obesity and dyslipidemia in women of childbearing age. Moreover, increase in orexin level was another independent risk factor for obesity. Intraventricular injections of different doses of orexin have been found to increase in a dose‐dependent manner [29]. Neuropeptide Y and orexin can be used as potential risk screening indicators for obesity in women of childbearing age. It is recommended to detect these two indicators in women preparing for pregnancy or during pregnancy. If the levels are significantly increased (even within the normal range), lifestyle interventions (such as dietary guidance and exercise intervention) can be carried out in advance to reduce the risk of obesity and related complications.

The present study found that the oxytocin level within the normal range in obese women was significantly lower than those in women of normal weight and was inversely correlated with BMI. It was found to be an independent protective factor for obesity in women of childbearing age. Oxytocin plays an important role in regulating energy metabolism, interacting with neurons in the hypothalamus that promote feeding (e.g., neuropeptide Y, orexin), as well as neurons (e.g., proopiomelanocortin) to inhibit feeding and reduce body weight [30]. Several studies also demonstrated that oxytocin was negatively correlated with the levels of triglycerides, total cholesterol, and low‐density lipoprotein cholesterol [31–33]. However, we failed to reach such conclusions in this study. Oxytocin can be used as an intervention target for obesity in women of childbearing age. In the future, the weight loss effect of low‐dose oxytocin supplementation therapy in women with postpartum obesity can be explored.

The results of this study showed that the proopiomelanocortin level within the normal range in obese women was significantly lower than that in women of normal weight and was negatively correlated with triglycerides level, suggesting that proopiomelanocortin was involved in the regulation of body weight and plasma lipids. Proopiomelanocortin is an appetite suppressor, a negative regulator of feeding behavior, energy metabolism and weight maintenance in the hypothalamus, and an antagonist of neuropeptide Y. Studies have shown that inactivating mutations in the proopiomelanocortin gene could lead to obesity [34], and increased expression of proopiomelanocortin‐processing products α‐MSH or enhanced activity of its receptor (MC4R) could lead to dose‐dependent appetite suppression, decreased food intake, weight loss, and increased glucose tolerance [35, 36].

One of the limitations of our study is that the study is limited by its cross‐sectional design and did not infer a causal relationship between circulating hormones levels and the development of obesity. Second, serial changes in serum endocrine hormone and hypothalamic neuropeptides need to be measured at different time points during the day according to the daily and pulsatile pattern of hormone release. Third, we did not systematically record the specific menstrual cycle phase. Since female sex hormones fluctuate significantly during the menstrual cycle, this limitation may affect the accurate interpretation of our study results. In future studies, we will optimize our participant data collection protocol by explicitly recording the menstrual cycle history of female participants and confirming their specific cycle phase at sampling. Last, the weight gain may be influenced by other various factors, such as mood, appetite, and activity. All of these may induce to potential biases. Since this study is a retrospective study, detailed lifestyle data were not fully recorded in the electronic medical records. In future prospective studies, structured questionnaires should be used to systematically collect lifestyle‐related potential confounders, thereby further controlling the impact of these confounding factors on the research results.

In conclusion, in the population of women of childbearing age, fluctuations within normal levels of a variety of endocrine hormones and hypothalamic neuropeptides played an important role in the regulation of body weight and plasma lipids. Relatively higher levels of neuropeptide Y and orexin increased the risk of obesity, while elevated oxytocin level decreased the risk. The biomarkers involved in this study can be focused on metabolic diseases related to women of childbearing age and can provide targets for subsequent disease mechanism research and clinical intervention; meanwhile, prospective studies are recommended to verify the causal relationship between fluctuations in endocrine hormones and hypothalamic neuropeptides within the normal range and the occurrence of obesity.

## Author Contributions

Yingying Tang and Fuman Du contributed to study conception and design; Xiukun Zhang, Lei Fan, Chengyuan Li, and Yudong Guo contributed to acquisition, analysis, and interpretation of data; Yingying Tang, Xiukun Zhang, Lei Fan, Chengyuan Li, and Yudong Guo contributed to draft the article; Fuman Du and Weimin Wang contributed to revision.

## Funding

This study was supported by the Heilongjiang Provincial Health Commission Scientific Research Project, 20220303060720; Heilongjiang Provincial Natural Science Foundation Project, LH2023H087.

## Disclosure

All authors read and approved the version to be published. Each author believes that the manuscript represents honest work.

## Conflicts of Interest

The authors declare no conflicts of interest.

## Data Availability

The data that support the findings of this study are openly available in Science Data Bank at https://doi.org/10.57760/sciencedb.29220, with the DOI 10.57760/sciencedb.29220.
